# Transdiagnostic irritability in youth: a clinical review

**DOI:** 10.1016/j.jped.2025.101457

**Published:** 2025-10-28

**Authors:** Luiza Magalhaes de Oliveira, Guilherme V. Polanczyk, Luisa Shiguemi Sugaya

**Affiliations:** Universidade de São Paulo (FMUSP), Faculdade de Medicina, Departamento de Psiquiatria, São Paulo, SP, Brazil

**Keywords:** Irritable mood, Emotional regulation, Child development

## Abstract

**Objective:**

Irritability is one of the most frequent concerns in pediatric mental health and a predictor of long-term psychopathology and burden. This article aims to provide a comprehensive review of non-episodic irritability along with evidence-based clinical recommendations for pediatric care professionals.

**Sources:**

A narrative review was conducted based on the literature from the last 10 years and additional key studies. Observational studies, randomized controlled trials, systematic reviews, and meta-analyses were considered.

**Summary of the findings:**

Non-episodic irritability is a transdiagnostic construct associated with several mental disorders. Research on etiological pathways suggests that irritability may involve distinct mechanisms, moderate heritability, and genetic associations with attention-deficit/hyperactivity disorder (ADHD), and depression. Clinical manifestations vary across different developmental stages, and distinguishing pathological from normative irritability may be challenging. Although there is no gold-standard measure, validated instruments are available. Despite recent advances, evidence-based treatment remains limited. Evidence supports psychosocial interventions that integrate parent- and child-focused components, as well as the use of stimulants for patients with comorbid ADHD. Antipsychotics are effective in reducing irritability in children with autism spectrum disorder. However, its use requires caution in non-autistic youth with irritability without a diagnosable disorder or with oppositional defiant disorder due to inconclusive efficacy and potential adverse effects.

**Conclusions:**

Although publications on non-episodic irritability have increased, the evidence remains relatively nascent. Further research is needed to advance the knowledge on underlying mechanisms, enhance clinical recognition, identify effective interventions, and support the development of new treatments, ensuring that irritability is adequately addressed in clinical practice.

## Introduction

Irritability is defined as proneness to anger that may impair an individual’s functioning [1]. Non-episodic irritability is defined as persistent and chronic, rather than the emergence of irritability in the context of a distinct episode. It is considered a transdiagnostic construct that occurs across many mental disorders, such as oppositional defiant disorder (ODD), disruptive mood dysregulation disorder (DMDD), anxiety disorders, attention-deficit/hyperactivity disorder (ADHD), and autism spectrum disorder (ASD). It manifests through temper outbursts that are disproportionate to developmental stages and/or through a chronic angry mood. Longitudinally, irritability is associated with an increased long-term risk for future psychopathology and negative outcomes, including internalizing and externalizing disorders, suicidality, poor peer functioning, poor physical health, and health service use [2,3].

Since the beginning of the century, advances have been made in understanding its phenomenology, course, and neurobiological foundations. Overcoming an important debate, evidence supports the distinction between episodic irritability - which occurs in a limited time frame and is often accompanied by other symptoms, such as changes in mood or sleep - and chronic/non-episodic irritability that persists over time [4]. To date, there is a broad consensus within the scientific community regarding the clinical relevance and conceptual validity of the non-episodic irritability construct, which is the focus of this review. Its external validity is supported by evidence of its longitudinal course, its predictive value for adverse outcomes and future psychopathology, as well as genetic studies indicating moderate heritability [1,2,5].

Despite the significant advances, the evidence-based management of irritability in youth remains limited, as few randomized clinical trials (RCTs) have investigated irritability as a primary outcome [1]. Specific pharmacological options are scarce and are usually extrapolated from studies on the treatment of neurodevelopmental disorders, such as ASD or ADHD. Clinicians frequently resort to antipsychotics to manage irritability, regardless of their deleterious adverse effects and the lack of psychosocial interventions. As irritability is one of the most common mental health concerns in youth for which families seek health services [6], it is essential that professionals involved in pediatric care be prepared to address this problem. In this review, the authors summarize available evidence and present key aspects related to diagnostic assessment and treatment of non-episodic irritability in children and adolescents, aiming to support an evidence-based clinical approach.

## Clinical presentation

Although irritability is highly correlated with different emotions, such as anger, rage, and frustration, and with pathophysiological mechanisms, such as emotion and mood dysregulation, it is considered a distinct construct. Irritability includes both a mood and a behavioral component, reflected by increased proneness to anger, increased sensitivity to provocation, and increased likelihood of behavioral outbursts. In addition, irritability stands out as it can be expressed in both externalizing and internalizing disorders [7]. As a trait, irritability is distributed continuously and occurs normatively in the population. In this context, delimiting pathological irritability in children can be challenging, as an optimal threshold has not been defined yet, and as different behaviors are expected in each developmental stage [1]. In preschool children, who are still developing inhibitory control and emotional regulation, irritability and temper tantrums are frequently seen. The maturation of self-regulation, through the improvement of executive functions, helps children tolerate frustration. Thus, in later childhood and adolescence, irritability tends to decrease in intensity and frequency [8].

Nevertheless, some children may present clinically significant irritability that deviates from age-normative patterns. Even in early ages, temper tantrums are not expected to occur on a daily basis. Also, temper tantrums with aggressive behavior or dysregulation followed by a prolonged recovery period (e.g., break or destroy things during a tantrum or tantrums until exhausted), or symptoms that occur in unexpected contexts (e.g., angry mood during fun activities or tantrums that occur out of the blue) may indicate pathological irritability [9].

Furthermore, regarding its clinical presentation, irritability has been classified into two dimensions: phasic irritability, characterized by acute temper outbursts, and tonic irritability, characterized by a persistent angry, grumpy, or grouchy mood. Although highly correlated, these dimensions may differ in genetics, comorbid conditions, and longitudinal course [10]. Future research will be important to elucidate distinct mechanisms involved in phasic and tonic irritability and inform clinical practice.

## Pathophysiological and developmental models

### Neuroscience frameworks

A translational model of chronic irritability has been proposed by Brotman et al. [4] According to this model, irritability is conceptualized as an aberrant response to frustrative non-reward and perceived threat. Reward-based dysfunctions may be explained by impairments in reward learning and by increased sensitivity to both reward receipt and omission [4]. In addition, increased sensitivity to threat may be characterized by a lower threshold for detecting and reacting to potential danger. Attention bias, a tendency to direct attention to angry faces, and hostile attribution bias, a tendency to interpret others' behaviors as threatening or hostile, can be observed in irritable children and are often associated with deficits in facial emotion processing [4].

Interestingly, a previous functional neuroimaging study suggested that neural mechanisms underlying irritability in children with anxiety disorders, DMDD, and ADHD may not differ by diagnosis, but rather through interactions between anxiety and irritability symptoms, showing different amygdala–medial prefrontal cortex connectivity during face emotion processing [11]. In contrast, Wiggins et al. found distinct patterns of amygdala activation during a face emotion labelling task when comparing children with DMDD and bipolar disorder (BD) [12]. Although these findings indicate different mechanisms underlying episodic and non-episodic irritability, it is important to notice that current evidence from neuroimaging studies is limited by the lack of reproducibility and small effect sizes, and remains inconclusive. A recent meta-analysis including 30 task-based fMRI studies found no neural activation convergence of irritability across neuro-cognitive functions related to emotional reactivity, cognitive control, and reward processing [13].

### Genetics

Twin studies suggest that the heritability of irritability ranges from 22 % to 51 % [1]. Also, irritability has genetic correlations with ADHD, antisocial behavior, and depression. More recent genetic research on the role of polygenic risk scores (PRS) indicated the association between irritability and ADHD PRS. Also, a longitudinal study identified distinct irritability trajectories, including: an early-onset and stable trajectory, associated with neurodevelopmental disorders, more prevalent in males and associated with ADHD PRS; and a late-childhood increasing trajectory, more common among females, related to later development of anxiety and depressive symptoms, and associated with both ADHD and depression PRS. Based on these findings, the hypothesis of distinct subtypes of irritability has been proposed [14], but still warrants further investigation through longitudinal genetic studies [1].

### Environmental contributions

Beyond its moderate heritability, irritability has also been associated with environmental adversities. Trauma is a well-established risk factor for a wide range of psychopathologies, and emerging evidence also links it to increased irritability [15]. In childhood, traumatic experiences may arise from various sources, including direct maltreatment or exposure to life-threatening events involving the child or close family members (e.g. serious illnesses or accidents). Despite the conceptual relevance of trauma to the development of irritability, few studies have specifically explored this association within a transdiagnostic framework.

Recently, a study investigating the effects of childhood trauma found that irritability was specifically associated with threat-related experiences, but not with deprivation-related adversities. Among children exposed to threat, higher trauma exposure was linked to increased levels of self-reported irritability [15]. Children who have experienced traumatic events also tend to exhibit heightened threat sensitivity, misclassification of emotional expressions, and attentional biases toward threat-related cues [16], patterns that overlap with the threat sensitivity mechanism observed in irritable children. In a study investigating children exposed to intimate partner violence (IPV), irritability was found to be associated with a greater total number of trauma types, increased exposure to interpersonal violence and emotional abuse, as well as higher severity of Post-Traumatic Stress Disorder (PTSD) symptoms [17].

Family dynamics may also exert a substantial influence on the emergence and persistence of irritability in children. Dysfunctional interactions between caregivers and children, especially in contexts marked by stress and emotional difficulties, can perpetuate disruptive behaviors and possibly irritability [8]. The management of irritable children can be particularly exhausting for parents, fostering a cycle of mutual escalation of emotional reactivity. In such contexts, caregivers commonly resort to coercive parenting strategies or even physical and psychological abuse, which may further exacerbate the child’s symptoms through gene–environment interactions [4].

These findings highlight the importance of clinicians investigating negative life events and traumatic experiences in the clinical evaluation of irritable children. They also underscore the need to assess family dynamics and caregivers’ mental health. Such an approach can guide more targeted interventions, focusing not only on the child but also on the family. It may also indicate the need to involve social support systems and child protective services when appropriate.

## Nosological perspectives on non-episodic irritability

A major discussion on the diagnosis of children with chronic/non-episodic irritability in youth began in the 1990s, when some researchers and clinicians hypothesized that non-episodic irritability accompanied by symptoms of hyperarousal, and without classic manic episodes, would be a developmental presentation of BD in childhood. This led to a sharp increase in the diagnosis of pediatric BD, particularly in the United States. In response to these concerns, Leibenluft et al. operationalized the construct of Severe Mood Dysregulation (SMD) [18] - defined by chronic and severe irritability - and conducted a series of studies comparing SMD and BD in terms of heritability, longitudinal course, and psychopathological correlates. The findings indicated that SMD was not a developmental phenotype that antedates BD. Rather, it was longitudinally associated with depression and anxiety. Thereafter, based on the SMD conceptualization, the diagnostic criteria for Disruptive Mood Dysregulation Disorder (DMDD) were operationalized, leading to its inclusion in the DSM-5 [19] with the aim of responding to the clinical needs of children with chronic and severe irritability.

To date, in the DSM-5, irritability is present in over 20 diagnostic categories [1,19]. Particularly, in DMDD and ODD, irritability is a central feature. But, in most of the disorders, including anxiety and mood disorders, as well as neurodevelopmental disorders, irritability is described as a nonspecific symptom or an associated feature supporting the diagnosis.

### Disruptive mood dysregulation disorder (DMDD)

According to the DSM-5, DMDD is characterized by severe recurrent temper outbursts that are grossly out of proportion to the situation, and persistent irritable mood between outbursts. Both symptoms must be present for at least 12 months and across at least two settings. Onset of symptoms must occur before the age of 10, and diagnosis can be made after 6 and before 18 years old. Also, DMDD and ODD cannot be diagnosed concurrently. When criteria for both disorders are met, the diagnosis of DMDD should be assigned instead [19]. A recent meta-analysis by Benarous et al. estimated a pooled prevalence of DMDD of 3.3 %. However, substantial heterogeneity was observed across studies, and when only studies that strictly adhered to all DSM-5 criteria were included, the prevalence dropped to 0.82 % [20].

It is relevant to note that the introduction of the DMDD in the DSM-5 raised several criticisms regarding its diagnostic validity. These include low interrater reliability among clinicians, concerns about limited population diversity in supporting studies, a lack of evidence for specific treatment approaches, and high rates of ODD comorbidity [21]. In light of these concerns, the ICD-11 did not include DMDD as a distinct diagnostic category but instead incorporated chronic irritability as a specifier within the diagnostic framework of ODD. Nonetheless, some have argued that, despite the diagnostic overlap, ODD includes additional behavioral dimensions (i.e., headstrong/defiant behavior and vindictiveness) that do not fully capture the core features of chronic irritability and recurrent temper outbursts.

Although more research is still necessary to address these questions - including cross-cultural studies and investigations about data-driven thresholds - a distinct diagnostic category such as DMDD sheds light on this important clinical problem and may help to address the significant impairment experienced by these youth [4].

### Oppositional defiant disorder (ODD)

ODD is characterized by three dimensions: a recurrent pattern of angry or irritable mood, defiant behavior, and vindictiveness. Although irritability is one of its diagnostic dimensions, it represents only a subset of a broader ODD clinical presentation, which may include psychopathological profiles that are different than those observed in youth with DMDD. Among children with ODD, only those with the most severe levels of irritability would meet diagnostic criteria for DMDD [22]. Furthermore, the functional impairment required for diagnosis differs between the two conditions: DMDD requires impairment in two contexts, whereas ODD requires impairment in only one. These distinctions highlight the need to evaluate the clinical presentation and functional impact of irritability when determining the most appropriate diagnosis.

### Attention deficit/hyperactivity disorder (ADHD)

ADHD and irritability frequently co-occur and share overlapping clinical and developmental features. Among ADHD patients, 25–50 % present symptoms of irritability. Conversely, approximately 60 % of youth with DMDD also meet criteria for ADHD [20]. A previous study conducted with a sample of irritable children with several disorders indicated that high ADHD symptoms were associated with significantly greater phasic irritability. Furthermore, data suggest that ADHD children with irritability may represent an ADHD subtype with distinct neurobiological and clinical features [23].

In addition, genetic studies suggest shared etiological pathways. ADHD PRS is associated with early irritability, indicating that irritability may reflect a clinical marker of higher genetic loading in children with ADHD [14]. Clinically, the presence of irritability in youth with ADHD is associated with greater severity and increased rates of comorbid affective disorders and ODD, reinforcing the need for early assessment and tailored interventions in this group.

### Autism spectrum disorder (ASD)

Irritability is a common reason for treatment-seeking among youth with ASD. Despite its clinical relevance, few studies have investigated mechanisms related to irritability in ASD. Instead, they have focused on related behaviors such as aggression, temper tantrums, or self-injurious behavior. It is possible that irritability in ASD may be related to pathophysiological processes that impair emotion regulation, which may be shared by other neurodevelopmental disorders such as ADHD (e.g., disrupted top-down regulatory mechanisms). However, ASD characteristics (e.g. difficulty identifying, distinguishing, and describing emotions, rigid thinking patterns, unusual reactions to sensory information) may also interfere with effective emotion regulation [24]. Considering the profound impact on child functioning and the burden on families and psychiatric services, the assessment and treatment planning of children and adolescents with ASD should consider the occurrence of irritability and related behavior.

### Other conditions associated with irritability

Non-episodic irritability in children has been associated with increased risk for future depression and anxiety [2]. Anxiety and irritability also share pathophysiological mechanisms, including heightened threat sensitivity and hostile attribution bias [4,11]. Moreover, episodic irritable mood is a common manifestation of depression in youth. In children and adolescents exposed to trauma, irritability may occur as part of the clinical presentation of post-traumatic stress disorder (PTSD), but can also appear in the absence of a full PTSD diagnosis.

## Assessment

Instruments for assessing childhood irritability are essential in research and clinical contexts, as they support both diagnostic assessment and longitudinal symptom monitoring. In the literature, irritability measures were often obtained from structured and semi-structured diagnostic interviews (e.g., Development and Well-Being Assessment [DAWBA] or the Schedule for Affective Disorders and Schizophrenia for School-Aged Children [K-SADS]), general psychopathology inventories (e.g., Child Behavior Checklist [CBCL]), or temperament rating scales. More recently, instruments that measure irritability specifically were developed. These tools are based on parent- and self-report, or clinical observation, and may vary according to the target age group, with scales available from early childhood through adolescence. In this context, instrument selection should consider available time, clinician training, target informant, and the child’s age.

Irritability measures with good psychometric properties include the Affective Reactivity Index (ARI), the Multidimensional Assessment of Preschoolers Study (MAPS) scales, and the irritability subscale of the Aberrant Behavior Checklist (ABC-I). The ARI [25] is a concise instrument that examines the child’s threshold for an angry reaction and the frequency and duration of angry feelings and behaviors. It has parent and self-report versions, which have been validated in clinical and non-clinical samples and translated into several languages, including Brazilian Portuguese [26]. Its clinician-rated version, the Clinician Affective Reactivity Index (CL-ARI) [27], shows promise for use in dimensional clinical assessment. The CL-ARI consists of 11 items, requires professional training, and includes three subscales: temper outbursts, irritable mood, and impairment.

The MAPS scales [28] have played an important role in the development of irritability assessment tools and remain promising for use across a wide age range. The MAPS Temper Loss (MAPS-TL) scale has underscored the dimensional nature of irritability in distinguishing normative from clinically significant patterns in preschool-aged children [9]. The scale has since been expanded to cover early childhood through adolescence, with age-specific versions.

The ABC is a parent-report tool developed to assess major behavioral concerns in individuals with neurodevelopmental disorders and is widely used to evaluate children with ASD. It is appropriate for use in children aged four years and older, making it particularly useful in early childhood assessments. The irritability subscale (ABC-I) includes components such as tantrums, self-injury, verbal outbursts, and negative affect [29].

In addition, observational tools provide useful information by offering quantifiable behavioral data. The Disruptive Behavior Diagnostic Observation Schedule (DB-DOS), a structured 50-minute observational protocol, evaluates behavioral regulation and problems in anger modulation within eliciting contexts [30].

An important consideration in the assessment of pediatric irritability is the frequent discrepancy between different informants. Reports from different caregivers, the child's self-report, and the clinician’s evaluation often diverge. However, these discrepancies should not be viewed as problematic, as they provide meaningful information about the child’s behavior across various contexts and relationships and how each informant perceives and interprets the child’s functioning [8]. Another important aspect to consider is the value of longitudinal monitoring, given that children often show substantial variability in symptom presentation over time, even in cases where symptoms are initially severe.

Currently, there is still no gold-standard instrument for the clinical assessment of irritability [1] and practical barriers, such as time and level of training needed, may limit their use. Therefore, in clinical practice, these tools should be applied as complementary resources within a broader diagnostic process, which may include a thorough assessment of irritability symptoms (including frequency, intensity, duration, and triggering events), occurrence of comorbidities, patient's functionality, family dynamics, and parenting practices.

## Treatment approaches

The number of treatment studies on irritability has been increasing. However, the evidence is still limited by the quality and number of RCTs that evaluate irritability as the primary outcome, particularly for non-ASD youth. Recently, Breaux et al. published the first systematic review and meta-analysis on non-episodic irritability that evaluated the efficacy of pharmacological and non-pharmacological interventions [31]. In total, there were 42 open trials and 59 RCTs. Among these studies, 80 studies focused on pharmacological interventions, 13 on nonpharmacological interventions, and 8 on combined interventions. Also, 84 included children with ASD or ASD plus ADHD, and only 17 studies included children with other diagnoses, including ADHD, disruptive behavior disorders (DBD), DMDD, and SMD.

Across all diagnoses, the overall pooled pre- to post-treatment effect on irritability was statistically significant (Hedges' *g* = 1.62). However, heterogeneity between studies was high. When analyzed separately, both nonpharmacological (*g* = 1.11) and pharmacological (*g* = 1.85) interventions were effective for reducing irritability. Antipsychotic medications provided the largest effect relative to all other medication classes and nonpharmacological interventions. However, treatment tolerability was not assessed in this meta-analysis. Thus, in clinical practice, it is important to weigh up the occurrence of adverse effects related to antipsychotic use, and nonpharmacological interventions should be considered as an initial treatment option.

Interestingly, this meta-analysis found large effect sizes among studies with ASD samples (*g* = 1.89) relative to studies with ADHD, DBD, DMDD, and/or SMD samples [31]. Methodological limitations should be considered, but these findings highlight the importance of considering the distinct clinical features of the ASD population and the risks of generalizing evidence from this group to youth without ASD.

On the other hand, transdiagnostic approaches in psychotherapy may be valuable in clinical settings, where children and adolescents often present with comorbidities and complex symptom profiles, rather than isolated conditions typically targeted in controlled research environments [32]. Integrating knowledge about the components of irritability to develop interventions that address a range of symptoms and diagnostic presentations may improve outcomes and functioning in a more comprehensive manner.

## Psychosocial interventions

Although few studies on psychosocial interventions have investigated irritability as the primary outcome, there is substantial evidence supporting their efficacy in children with disruptive and aggressive behaviors. Psychosocial treatments are commonly categorized as either parent- or child-focused interventions. However, they can also be delivered through integrated approaches that combine both components. Age is a relevant factor in treatment planning, with evidence suggesting that parent management training tends to provide better outcomes in younger children, while cognitive-behavioral therapy (CBT) appears more effective in adolescents. Nonetheless, both approaches remain valuable across developmental stages and can be adapted according to the child’s age and needs. Additionally, therapeutic planning should consider service availability as well as the patient's specific clinical characteristics.

### Parent management training

Parent Management Training (PMT) is a psychosocial intervention that teaches caregivers strategies to address maladaptive behaviors through positive reinforcement, clear and assertive communication, consistent behavioral management, and the promotion of prosocial behaviors. Given the role of familial dynamics and the interactions between child irritability, inconsistent responses, and excessive accommodation, empowering parents with effective tools can foster the emergence of more adaptive behavioral patterns in children. PMT has shown good evidence for reducing disruptive and aggressive behaviors in children, with several structured programs available, including Helping the Noncompliant Child, Triple P – Positive Parenting Program, and The Incredible Years. Although published RCTs indicated that PMT is effective in reducing irritability [33, 34, 35], more studies are needed to investigate the efficacy of PMT for reducing irritability across different populations [36].

### Child-focused therapies

Child-focused therapies aim to promote the development of emotion regulation and problem-solving skills, and preliminary studies suggest potential benefits in reducing irritability in youth. CBT has been primarily studied in the context of externalizing, disruptive, and antisocial behaviors [1]. A meta-analysis of CBT for anger-related problems in children and adolescents reported a moderate effect size, supporting its applicability to irritability-related presentations [37]. More recently, interventions specifically targeting irritability symptoms were developed. An adapted version of dialectical behavior therapy (DBT) for children with DMDD demonstrated positive outcomes on reducing symptom severity [38]. Interpersonal psychotherapy tailored to DMDD symptoms also showed improvement in global clinical severity, although no significant changes were observed on standardized irritability measures [39]. In an RCT of an Interpretation Bias Training (IBT) targeting hostile attribution bias, children in the intervention group would more frequently interpret ambiguous facial expressions as happy. However, it did not show a reduction in irritability [40]. Further research is needed to clarify the role of mechanism-focused interventions in the treatment of irritability in youth.

### Transdiagnostic programs

The Modular Approach to Therapy for Children with Anxiety, Depression, or Conduct Problems (MATCH) and the Unified Protocol for Transdiagnostic Treatment of Emotional Disorders in Children (UP-C) are transdiagnostic CBT interventions that have been applied to a range of mental health conditions. Although not originally designed to target irritability, evidence suggests that both approaches may be beneficial in reducing these symptoms [41,42]. In line with a transdiagnostic perspective, a recent exposure-based CBT protocol combined with PMT was developed specifically to target irritability mechanisms and has demonstrated reductions in irritability and temper outbursts among children diagnosed with DMDD [35].

## Pharmacological treatments

Pharmacological treatment may be indicated in more severe cases or when psychosocial interventions fail to produce significant improvement in irritability. It is essential to consider comorbid neurodevelopmental disorders when selecting the appropriate drug.

Most studies on antipsychotics have been conducted in populations with ASD, and caution is warranted when extrapolating these findings to non-ASD youth. For irritable children with ASD, risperidone and aripiprazole show good results [43]. However, to date, there are no high-quality RCTs evaluating the use of antipsychotic monotherapy for irritability in non-ASD children with preserved cognitive functioning.

In children with comorbid ADHD, treating ADHD core symptoms is recommended [8], as studies have shown that irritability may improve with the use of stimulants. In aggressive children with ADHD, combined treatment with stimulants and behavioral therapy has been associated with symptom improvement, and non-responders may benefit from augmentation with risperidone or divalproex [44]. Nevertheless, the number of children examined in the augmentation phase was small, and this approach cannot yet be considered an evidence-based recommendation.

The use of an antidepressant has been investigated in a small trial with children and adolescents who met criteria for SMD. Citalopram, added to a previously optimized dose of methylphenidate, showed a significant reduction in irritability when compared to methylphenidate and placebo [45]. However, no differences in functional impairment were observed.

Adverse effects must be carefully considered when prescribing pharmacological treatments in pediatric populations, with particular concern regarding antipsychotics. Harmful effects such as metabolic and hormonal disturbances, neurological or cardiovascular side effects must be considered [46]. In summary, evidence supports the use of antipsychotics (risperidone and aripiprazole) for irritability in ASD youth. When ADHD is comorbid, stimulant treatment optimization may reduce irritability. In other diagnostic groups, pharmacological strategies should be approached cautiously due to limited data. These findings underscore the importance of further high-quality research to guide pharmacological decisions in pediatric irritability.

## Conclusion

Recent evidence shows that, despite growing recognition of the importance of studying irritability and the exponential increase in publications on the topic, many key questions remain unanswered. Advancing the understanding of the mechanisms underlying the manifestation of irritability is a promising path, as it may provide a theoretical foundation for further discoveries. These could have significant clinical implications, such as enabling the early identification of pathological irritability, improving the prediction of clinical trajectories, and guiding the development of more effective treatments. More RCTs are needed to evaluate the impact of different interventions on this population.

From a nosological perspective, ongoing discussions about the categorization of irritability highlight the need for further refinement of diagnostic criteria, particularly for DMDD. Building a valid and reliable diagnostic framework is essential to better identify impaired children and to guide appropriate public health strategies. Important questions that remain include: What mechanisms are shared across different diagnoses involving irritability, and which are specific to each condition? Do different mechanisms of irritability require different clinical approaches, or would a more integrated model be more effective? Is there more than one subtype of chronic irritability?

Despite the many open questions, some practical insights have already emerged. It is strongly recommended that irritability be assessed when present in clinical settings, as it is associated with significant impairment and carries a well-documented risk for future psychopathology. In terms of treatment, there is growing support for integrative approaches that involve both the child and caregivers as a first-line strategy. Additionally, current evidence supports the use of ADHD medications in youth with comorbid ADHD and cautions against the widespread use of antipsychotics in non-autistic irritable children.

Taken together, recent advances in transdiagnostic irritability research are providing an important foundation for further discoveries in the field. Continued high-quality research is needed to translate these scientific contributions into more targeted and effective care for this population.

## Funding sources

This project did not receive direct funding.

## Data availability

The data that support the findings of this study are available from the corresponding author.

## Conflicts of interest

Guilherme V. Polanczyk has served as a speaker and/or consultant to Abbott, Ache, Adium, Apsen, EMS, Libbs, Medice, Takeda, and receives authorship royalties from Manole.
